# Plant Seed Mucilage as a Glue: Adhesive Properties of Hydrated and Dried-in-Contact Seed Mucilage of Five Plant Species

**DOI:** 10.3390/ijms22031443

**Published:** 2021-02-01

**Authors:** Agnieszka Kreitschitz, Alexander Kovalev, Stanislav N. Gorb

**Affiliations:** 1 Department Functional Morphology and Biomechanics, University of Kiel, Am Botanischen Garten 1–9, D-24118 Kiel, Germany; akovalev@zoologie.uni-kiel.de (A.K.); sgorb@zoologie.uni-kiel.de (S.N.G.); 2 Department of Plant Morphology and Development, Institute of Experimental Biology, University of Wrocław, Kanonia Street 6/8, 50-328 Wrocław, Poland

**Keywords:** cell wall, diaspores, seed mucilage, Linum, Plantago, Ocimum

## Abstract

Seed and fruit mucilage is composed of three types of polysaccharides—pectins, cellulose, and hemicelluloses—and demonstrates adhesive properties after hydration. One of the important functions of the mucilage is to enable seeds to attach to diverse natural surfaces. Due to its adhesive properties, which increase during dehydration, the diaspore can be anchored to the substrate (soil) or attached to an animal’s body and dispersed over varied distances. After complete desiccation, the mucilage envelope forms a thin transparent layer around the diaspore creating a strong bond to the substrate. In the present study, we examined the mucilaginous seeds of six different plant taxa (from genera Linum, Lepidium, Ocimum, Salvia and Plantago) and addressed two main questions: (1) How strong is the adhesive bond of the dried mucilage envelope? and (2) What are the differences in adhesion between different mucilage types? Generally, the dried mucilage envelope revealed strong adhesive properties. Some differences between mucilage types were observed, particularly in relation to adhesive force (*F_ad_*) whose maximal values varied from 0.58 to 6.22 N. The highest adhesion force was revealed in the cellulose mucilage of *Ocimum basilicum*. However, mucilage lacking cellulose fibrils, such as that of *Plantago ovata*, also demonstrated high values of adhesion force with a maximum close to 5.74 N. The adhesion strength, calculated as force per unit contact area (*F_ad_/A*_0_), was comparable between studied taxa. Obtained results demonstrated (1) that the strength of mucilage adhesive bonds strongly surpasses the requirements necessary for epizoochory and (2) that seed mucilage has a high potential as a nontoxic, natural substance that can be used in water-based glues.

## 1. Introduction

Mucilage of plant seeds and fruits (diaspores) is a polysaccharide complex consisting mainly of pectins and hemicelluloses, but also containing cellulose fibrils, which interact with each other by various types of bonds, forming a spatial network [1,2,3,4]. Diaspore mucilage can be classified based on its chemical composition into three main types: pectic, cellulose and hemicellulose. Pectic mucilage is typical of plant representatives from the genus Linum. Cellulose mucilage contains cellulose fibrils acting as a micro-/nanoscale “skeleton”, which anchor the mucilage to the seed surface and is characteristic of representatives from families such as, e.g., Asteraceae, Brassicaceae or Lamiaceae [5,6,7,8,9]. Hemicellulose-rich mucilage was recently defined in some species of the genus Plantago (*P. ovata* and *P. psyllium*)*,* in which the mucilage envelope can be dominated by hemicelluloses such as, e.g., arabinoxylan [10].

Mucilage production by diaspores is known as myxodiaspory and is particularly common and well developed in plants inhabiting steppes, semi-deserts or disturbed habitats, since the ability to produce the mucilaginous envelope has an adaptive advantage in arid environments [5,6,8,9,11,12]. Due to its capability to absorb and retain water, the mucilage stimulates seed germination by providing a proper microenvironment, facilitates anchoring of the diaspore by adhering it to the ground, and aids in long-distance dispersal by attaching to bird feathers or animal fur [10,11,12,13]. Regardless of the mucilage type, the adhesive properties can have some selective advantages. Adhesive, mucilaginous seeds can be deposited between feathers/fur or on the body of migrating animals and transported over shorter or longer distances [11,14,15]. Along with long distance dispersal, the mucilage can act as antitelochory factor, which means that the diaspore can be anchored to the ground and begin germination [16,17].

Mucilage secreting cells (MSCs), which are an integrative part of the seed/fruit coat, are responsible for mucilage production. The mucilage is released just after hydration in a gel-like capsule surrounding the diaspore [6,8]. This mucilaginous envelope demonstrates some specific physical properties, such as low friction and enhanced adhesion, both depending on the water content and chemical composition of the mucilage [18,19].

To characterize physical properties of plant material samples, diverse biomechanical studies have been conducted. Some micromechanical investigations have been focused on the behavior of tissues, individual plant cells or plant cell walls and interactions between their components [20,21,22,23]. The cytomechanical methods used in the cell wall studies include atomic force microscopy (AFM), microindentation and cellular force microscopy [23]. In various micromechanical experiments, cellulose, cellulosic fibers, wood, entire shoots or plant fragments have been examined under mechanical loading [21,24,25,26,27,28,29,30]. In our previous studies, we measured pull-off forces of the hydrated mucilage envelope in contact with a glass surface and the obtained maximal adhesion reached 91 mN [18,19].

Due to its hydration and strong chemical binding properties, the seed mucilage has found application as a binding agent in pharmaceuticals and as a thickening component in the food industry [31,32]. Pectins, the main mucilage component, play an important role as a gelling agent and stabilizer. The adhesive strength and viscosity of some seed mucilages can be higher in comparison to some synthetic polymers, such as hydroxyl-propyl-methyl-cellulose [33].

In our previous studies, we examined mucilage adhesion in relation to the hydration level. We considered the first stage of possible contact formation, when the seed with its mucilage envelope is attached to an animal as the dispersal agent [18,19]. In the present study, we were interested in the adhesive properties of the mucilage envelope that dried out remaining in contact established in a hydrated state. It was expected that adhesive properties would vary between different mucilage types. In our present study, we asked two main questions: (a) how strong are the adhesive properties when the mucilage envelope has dried out in contact with glass surface, and (b) how does adhesion of the mucilage envelope differ between diverse mucilage types?

## 2. Material and Methods

The plant material used in the experiments included seeds of six different plant taxa: *Linum usitatissimum* L. (flax)*, Lepidium sativum* L. (garden cress)*, Ocimum basilicum* L. (basil), *Plantago lanceolata* L. (plantain), *Plantago ovata* L. (blond plantain), *Salvia hispanica* L. (Chia). The seeds were obtained from the commercial supplier (Covered Marked, Wrocław, Poland) and from the wild populations (in case of *Plantago lanceolata*) collected in Wrocław-Zakrzów (leg. et det. A. Kreitschitz). For the experiments, only mature seeds were used. We also performed measurements for isolated seed coats (husk) of *Plantago ovata* (commercial supplier—LOGICO.PL, Damian Darowski, Domaszkowice, Nysa, Poland). The husk of *P. ovata* contains only mucilaginous cells and could be used as a kind of separated natural “glue” spread as a uniform layer on the substrate (glass).

### 2.1. Morphology of the Mucilage Envelope

To characterize the mucilage envelope and to detect its basic components, we performed following staining reactions. Staining with 0.1% aqueous solution of ruthenium red (Sigma-Aldrich) aided in detection of pectins [34]. The presence of crystalline cellulose was tested in a polarized light microscope (Zeiss LSM 700) (Zeiss, Jena, Germany) [35]. Images were taken using a light microscope equipped with digital camera. To detect the presence of starch in the mucilage of *Ocimum basilicum,* the solution of potassium iodide with iodine (IKI) in water was used: it stained starch grains violet to dark violet [34].

### 2.2. The Pull-Off Force Measurements of Dehydrated Mucilage Envelope

The material tester Zwick Roell z 0.5 (Zwick GmbH & Co. KG, Ulm, Germany, Type: BT1-FR0.5TN.D14) was used in the experiment. It allowed us to conduct experiments in established, constant conditions. The results of the measurements were automatically recorded and processed by Zwick Roell software.

**Figure 1:**
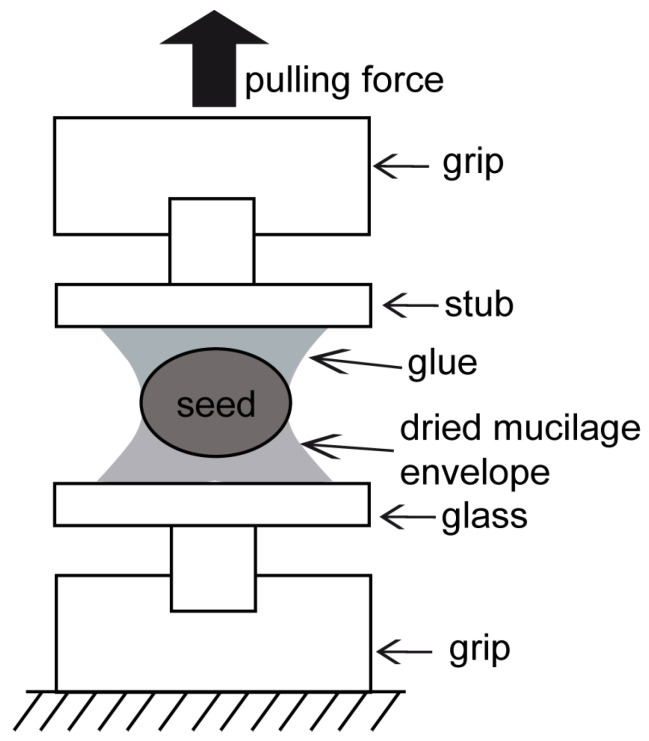
Probe fixation in pull-off experiments. The sample with the seed was placed between two grips: the lower one was motionless and the upper one moved upwards. The lower seed surface was attached to the aluminum SEM stub by the dried mucilage envelope. The upper (mucilage free) seed surface was glued to the SEM stub by the commercial glue. The upper grip moved up and stopped when the seed was detached from the glass slide.

For every taxon, 15 individual seeds were hydrated for 30 min, in order to enable formation of the mucilage envelope. The seeds were then put on a glass slide and left to dry out for at least 24 h. After that time, the seeds had strongly adhered to the glass by the dried-out mucilage envelope. The contact area between the mucilage envelope and the glass substrate was measured using a stereoscopic microscope, Leica M205 A (Leica Mikrosysteme, Wetzlar, Germany). The contact area was used for the calculation of adhesion strength. For the adhesion measurements, two surfaces were used: a glass slide with an attached (by mucilage) seed and a SEM stub with a round cover glass bonded with commercial glue (Cyanoacrylate adhesives: 5925 ergo(R) Elastomer–Kisling GmbH, Bad Mergentheim, Germany). The glass slide with the attached seed was fastened to the lower pincer grip, and the SEM stub was fastened to the upper pincer grip (Figure 1) in the Zwick material tester. The upper (free) seed surface was attached to the SEM stub with a commercial glue (BONDIC GmbH, Köln, Germany) (Figure 1). The upper grip was moved up with the speed of 2 mm/s, until the seed was detached from the glass slide. The force–distance curves were continuously recorded during the platform motion.

The average time needed to perform biophysical experiment and measurement with Zwick (including the following steps: sample fixation in Zwick, setting up/checking the probe parameters, measurement and saving the data) ranged between 20–25 min. The measurement of the pull-off force for the individual sample took from few to several seconds (9–max 120 s).

As a control, 15 dry seeds of each studied taxon were fixed to the glass slide with a drop of UHU glue (all-purpose adhesive, liquid-UHU GmbH & Co. KG, Bühl, Baden, Germany). Prior to adhesion force measurements, images of the contact area between the seed and the glass substrate were taken using a stereoscopic microscope as mentioned above. After the measurements, a second image was taken, to visualize the area where the glue was peeled away from the glass. The area was estimated with AxioVison SE64 Rel. 4.8 software (Zeiss, Jena, Germany).

For the adhesion force measurements of *Plantago ovata* husk mucilage, 15 samples were also used. A round cover glass (12.5 mm in diameter) was adhered to a SEM stub with the commercial glue (Cyanoacrylate adhesives: 5925 ergo(R) Elastomer–Kisling GmbH, Bad Mergentheim, Germany). The seed husks were hydrated for 30 min to create the mucilage. Then, a 60 µL drop of mucilage material was put on the stub with the attached round cover slip and pressed with a second stub with an attached cover slip. The samples were air-dried for 24 h. As a control sample, two SEM stubs with cover slips were connected using a 60 µL droplet of commercial UHU glue (all-purpose adhesive, liquid-UHU GmbH & Co. KG, Bühl, Baden, Germany; contains–ethanol, methyl acetate) and air-dried for 24 h. For the measurement, a sample was put between two grippers of the Zwick tester, and the measurement was carried out as described above. The time needed for the measurements of individual samples using Zwick was ranging between 20–25 min.

Scanning electron microscopy (SEM) images were taken of all seed types of studied plant taxa, *P. ovata* husk and UHU after force measurements, in order to analyze the mucilage and UHU glue structure at the fracture zone. The samples were coated with gold palladium (film thickness 10 nm) using a Leica EM SCD 500 High Vacuum Sputter Coater (Leica Microsystems GmbH, Wetzlar, Germany), and visualized in SEM Hitachi S-4800, Hitachi High-Tech. Corp., Tokyo, Japan).

### 2.3. Young’s Modulus Determination

Young’s moduli of both UHU glue and husk mucilage from the seeds of *P. ovata* was determined. *P. ovata* was chosen, as we knew we were able to collect a sufficient amount of mucilage from this species for the experiment. UHU glue was used as a control. Defect-free stripes were prepared from the two materials and pulled with 2 mm/min velocity in the Zwick tester until 5% stretched. For calculation of the Young’s moduli, the values of maximum stiffness and cross-sectional area were used. The area was measured using a VR-3000 3D Measurement Microscope.

### 2.4. Statistics

The statistical analysis was done using Sigma-Plot (Systat Software Inc, San Jose, CA, USA). The results were analyzed using the non-parametrical Kruskal–Wallis one-way ANOVA by ranks test (*p* < 0.05) and multiple comparison procedures (Dunn’s Method, *p* < 0.05), because the normality test failed.

## 3. Results

### 3.1. Mucilage Morphology and Its Basic Components

**Figure 2:**
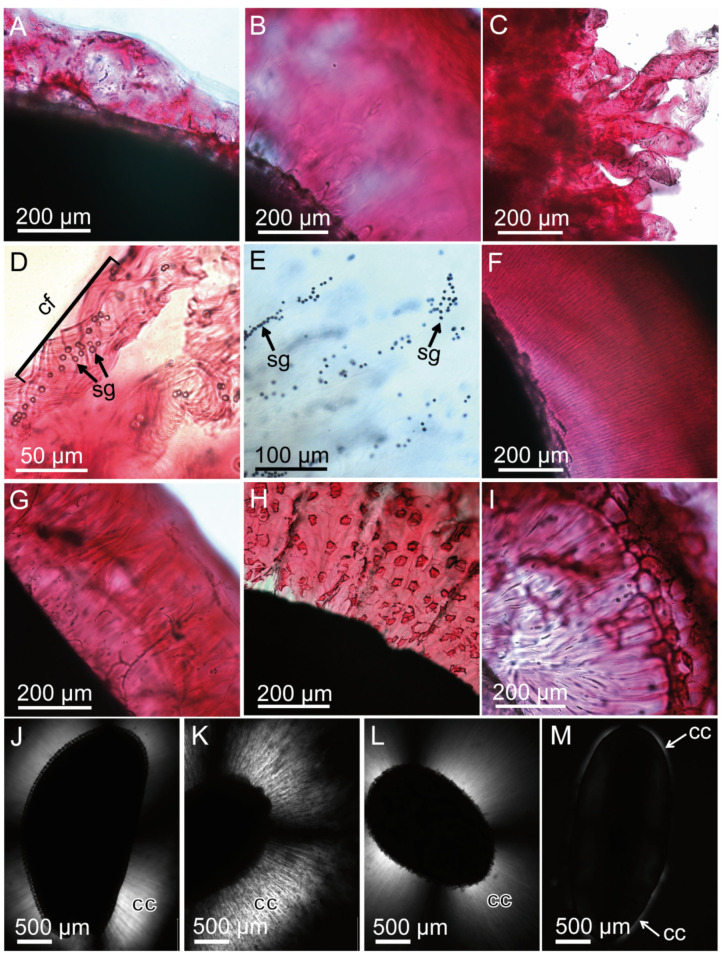
Mucilage morphology presented using optical microscopy. (**A**–**I**) Staining with ruthenium red. Characteristic purple color indicates the presence of pectins in the mucilage; (**A**) *L. usitatissimum*; (**B**) *L. sativum*; (**C**–**E**) *O. basilicum*; (**D**) Characteristic “mucilaginous tubules” with spirally coiled cellulose fibrils (cf) with enclosed inside starch grains (sg) in the mucilage of *O. basilicum*; (**E**) staining of starch grains with IKI. The grains have dark coloration; (**F**) *S. sclarea* mucilage with delicate, radially spread cellulose fibrils; (**G**) *P. lanceolata*; (**H**,**I**) *P. ovata* seed mucilage (**H**) and husk mucilage (**I**); (**J**–**M**) detection of crystalline cellulose (cc) (polarized light microscope) in the mucilage envelope; (**J**) *L. sativum*; (**K**) *O. basilicum*; (**L**) *S. sclarea*; (**M**) *P. lanceolata*.

In studied taxa, the mucilage envelope forms a gel-like capsule around the seed within a few minutes after hydration. Based on our previous studies [18,19] and staining reactions applied in the present study, we were able to describe the basic components and the main types of the mucilage (Figure 2A–D,F–I). The staining by ruthenium red revealed the presence of pectins, which formed the main mass of the mucilage envelope. The mucilage of *Linum usitatissimum* belongs to the pectic type. The cellulose mucilage is typical for *Lepidium sativum*, *Ocimum basilicum* and *Salvia hispanica* (Figure 2J–L)*,* and the presence of cellulose in the mucilage of *Plantago lanceolata* was revealed in the layer close to the seed surface (Figure 2M). Mucilage of *O. basilicum* had a characteristic “tubular” form with clearly visible, spirally coiled cellulose fibrils and starch grains (Figure 2C–E).

The presence of cellulose fibrils with crystalline regions was verified and visualized using polarized light microscope (Figure 2J–M). The crystalline cellulose was present in the mucilage of *L. sativum* (Figure 2J), *O. basilicum* (Figure 2K) and *S. hispanica* (Figure 2L) and in a very narrow region close to the seed surface in *P. lanceolate* (Figure 2M). The mucilage of *P. ovata* represents a hemicellulose-rich type without cellulose fibrils (see also [36]). The presence of starch grains in the mucilage of *O. basilicum* was detected (Figure 2E).

### 3.2. Measurements of Pull-Off Forces of the Dehydrated Mucilage Envelope

**Figure 3:**
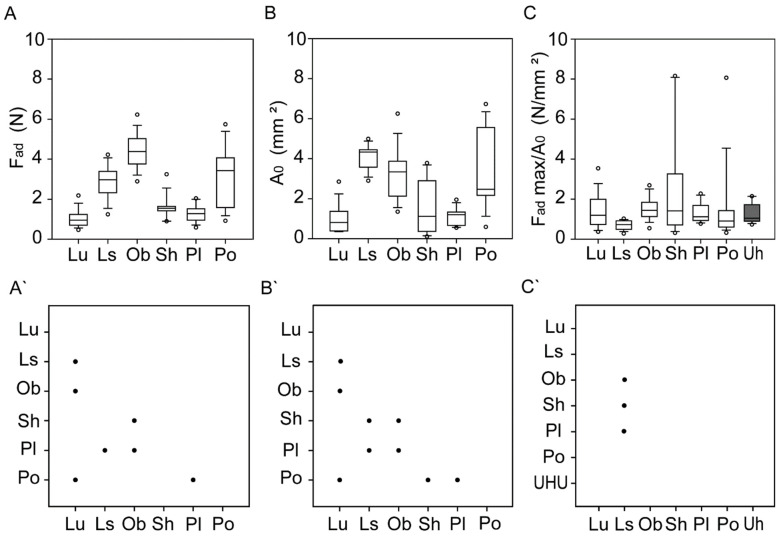
(**A**–**C**) Adhesion force (**A**), contact area (**B**) and adhesive strength (**C**) of the seed mucilage dried out in contact. (**A’**–**C’**) Multiple comparison graphs of the adhesion force (**A’**), contact area (**B’**) and strength force (**C’**) of the seed mucilage. Statistically significant differences are marked with dots. Abbreviations: Lu—*L. usitatissimum*, Ls—*L. sativum*, Ob—*O. basilicum*, Sh—*S. hispanica*, Pl—*P. lanceolata*, Po—*P. ovata*, Uh—UHU glue. In the box-and-whisker diagrams, the bottom and top of the box are the 25th and 75th percentiles, the line inside the box is the median; the ends of the whiskers are the 10th and 90th percentiles.

The studied plants can be divided into two groups with statistically significant differences (Figure 3A,A’). The first group contains *L. usitatissimum*, *P. lanceolata* and *S. hispanica*, and the remaining species, *L. sativum*, *O. basilicum* and *P. ovata,* were allocated to the second group. The mean *F_ad_* (adhesion force) values for group one ranged from 1.01 to 1.59 N. In the second group, the mean *F_ad_* values were more than three times higher (e.g., *O. basilicum* with *F_ad_* = 4.44 N) (Table 1).

The same situation was observed for the mean *A*_0_ (contact area) values (Figure 3B,B’). The mean contact area was relatively small in the first group ranging from 0.98 to 1.57 mm^2^. In the second group, the values were three to four times higher (Table 1).

In the case of adhesive strength (*F_ad_/A*_0_), the mean results were similar in representatives of both groups (Figure 3C,C’, Table 1). The statistically significant differences were observed only for *L. sativum,* which differed from the other three (*P. lanceolata*, *O. basilicum*, *S. hispanica*).

It was observed that during individual measurements, the pulling force increased gradually until it reached the maximum—the point when the seed with some mucilage residue was detached from the glass substrate. The pull-off force (*F_ad_*) for dried mucilage in all seeds varied from 0.58 to 6.22 N. The highest maximal value of *F_ad_* was measured in the cellulose mucilage of *O. basilicum*—6.22 N—which is considerably higher than the maximal *F_ad_* value of 2.8 N measured for the *O. basilicum* seeds embedded in the UHU glue. The lowest maximal value of *F_ad_* was that of *P. lanceolata*—2.03 N.

The contact area varied from 0.11 mm^2^ for *S. hispanica* to 6.73 mm^2^ for *P. ovata*. The mean contact area for seeds glued with UHU varied from 0.86 mm^2^ for *S. hispanica* to 5.49 mm^2^ for *L. usitatissimum*. The highest maximal value of adhesive strength, *σ*_c_ = *F_ad_/A*_0_, was observed for *S. hispanica* (8.15 N/mm^2^), and the smallest for *L. sativum* (1.02 N/mm^2^) (Table 1).

### 3.3. Adhesion Strength of P. ovata Husk Mucilage

**Figure 4:**
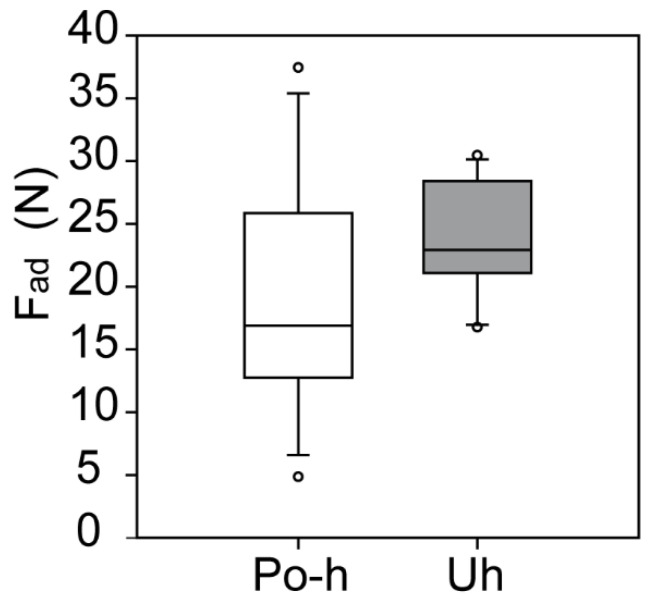
Adhesion force of *P. ovata* husk mucilage and UHU glue control sample. Abbreviations: Po-h—*Plantago ovata* husk mucilage, Uh—UHU glue. In the box-and-whisker diagram, the bottom and top of the box are the 25th and 75th percentiles, the line inside the box is the median; the ends of the whiskers are the 10th and 90th percentiles.

Because the contact area of the tested samples was constant, 122.65 mm^2^ for this experiment, the adhesion strength values were simple to obtain. The adhesion strength of dried-out *P. ovata* husk mucilage ranged between 0.04 and 0.31 N/mm^2^. The UHU glue control samples had adhesion strengths that varied from 0.14 to 0.19 N/mm^2^. However, the maximal adhesion force of UHU (30.4 N) was lower than the *P. ovata* (37.4 N) husk mucilage (Figure 4).

### 3.4. Mucilage Structure in Fractures after the Pull-Off Measurements

**Figure 5:**
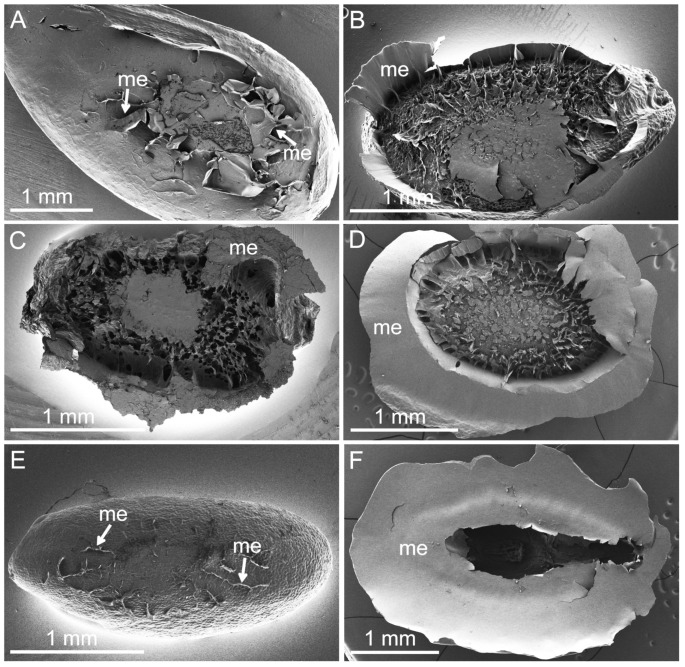
Seed mucilage structure in the fractures of the adhesive layer after pull-off measurements. (**A**) *L. usitatissimum* seed with small remains of the mucilage envelope (me, arrows) on the seed surface; (**B**) *L. sativum*; (**C**) *O. basilicum*; (**D**) *S. hispanica*; (**E**) *P. lanceolata* and visible small fragments of the mucilage envelope (me, arrows); (**F**) *P. ovata*. The mucilage envelope after drying formed a thin, transparent layer. After measurements, when the seed was detached from the glass, that thin layer stayed partially attached to the seed, which is visible as small (**A**,**E**) or larger fragments on its surface (**B**–**D**,**F**).

After hydration, mucilage envelope formation and drying out, the mucilage envelope formed a thin, transparent layer around the seed, which firmly attached the seed to the glass surface. The seed was then detached from the glass, together with the remaining mucilage envelope fragments (Figure 5). In *L. usitatissimum* and *P. lanceolata,* only small pieces of mucilage were found attached to the seed surface (Figure 5A,E). Interestingly, these two taxa also produced smaller amounts of mucilage in comparison to the other seeds studied, and in the other taxa, larger fragments remained attached to the seed surface (Figure 5B–D,F). The main part of the mucilage envelope remained attached to the glass surface after the pull-off experiment in all cases.

**Figure 6:**
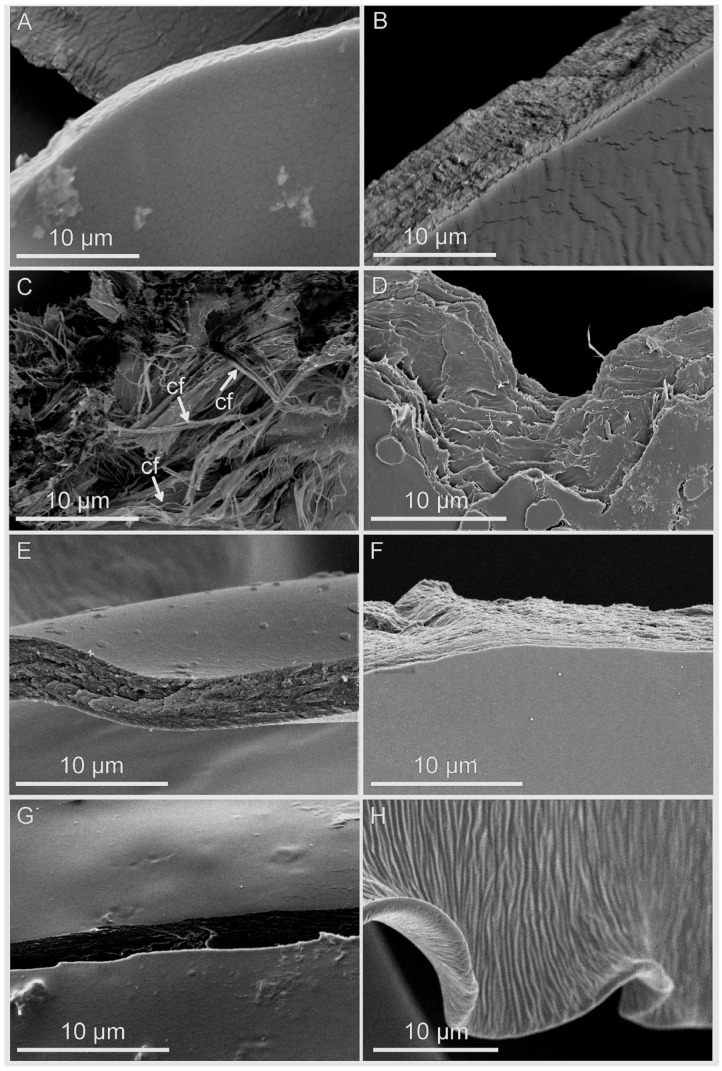
Mucilage envelope fractures after pull-off tests. (**A**) *L. usitatissimum*; (**B**) *L. sativum.* In both taxa, homogenous edges are visible; (**C**) *O. basilicum*, visible cellulose fibrils (cf) protruding from the mucilage envelope; (**D**) *S. hispanica*, fragment of the mucilage with visible lamellar structure; (**E**) nearly homogeneous mucilage layer of *P. lanceolata*; (**F**) *P. ovata* seed mucilage; (**G**) *P. ovata* husk mucilage; (**H**) wrinkled UHU glue layer.

The segments of the mucilage envelope differed in structure (Figure 6A–G) in comparison to the commercial glue sample (UHU), which resembled the fracture of an elastic polymer (Figure 6H). Mucilage fractures of *L. usitatissimum*, *L. sativum* and Plantago seeds demonstrated a homogeneous structure (Figure 6A–B,E–G), as opposed to the pieces of cellulose mucilage of *O. basilicum,* which showed heterogeneous structures with many torn fibrils of varying direction. The mucilage of *O. basilicum* and *S. hispanica* had lamellar organizations (Figure 6C,D) with cellulose fibrils embedded in the envelope. Additionally, in case of *O. basilicum,* starch grains were immersed in the mucilage.

### 3.5. Elasticity Modulus and Strain Energy Density of P. ovata Dried Husk Mucilage

The Young’s modulus of the dried-out husk mucilage of *P. ovata* was 423 ± 95 MPa, which is comparable to many stiff polymeric materials, e.g., Teflon [37], and one order of magnitude higher than the Young’s modulus of polymerized UHU glue, 45.4 ± 21.5 MPa. For the known Young’s moduli, *E*, and adhesion strength, *σ_c_*, the strain energy density, *W*, can be calculated as [38]: $W=\;\frac{\sigma_{c}^{2}h}{2E}$, where *h* is the elastic film layer thickness, which connects the two rigid stubs. In our pull-off experiments, with a layer of dried-out husk mucilage of *P. ovata* or a layer of UHU glue between two glass surfaces, the failure of separation always took place at the interface between the elastic film and the glass. The strain energy density for the adhesive failure of the husk mucilage on the glass was 2.74 ± 0.64 mJ/m^2^, whereas the strain energy density for the adhesive failure of UHU glue on the glass was 36.5 ± 6.1 mJ/m^2^. A high correlation coefficient between adhesion strength and strain energy density for individual measurements on husk mucilage, R = 0.89 (N = 16), allows us to assign the variation of pull-off force to strain energy density in individual samples.

## 4. Discussion

In the present work, we demonstrated the strong adhesive properties of the dried seed mucilaginous envelope. The maximal pull-off force (*F_ad_*) measured for different types of mucilage varied from 2.03 to 6.22 N and exceeded the adhesive properties of the commercial glue used as a control sample. The results of adhesive strength (*F_ad_/A*_0_) measurements were similar between different mucilage types, leading us to conclude that, in general, the mucilage envelope possesses strong adhesive properties.

The adhesive properties of the seed/fruit mucilage envelope are known to be beneficial for plants by aiding in diaspore transport [13]. The ability of seeds/fruits to travel by means of adhesion can be crucial in the dispersion success of many plants. It allows the plant to be introduced into new habitats, which leads to an increase in its area of distribution. The diaspore adhesion to the ground can also increase its survival chance, since after hydration, mucilage formation and anchorage to the soil, the diaspore can begin germinating [7,39].

Furthermore, due to its adhesive properties, the mucilage has also been utilized in pharmaceuticals as a thickening, binding, disintegration, suspension, emulsifying, stabilizing and gelling agent [33].

### 4.1. Polysaccharide Structure Can Influence Adhesive Properties of the Mucilage Envelope

The adhesive properties of polysaccharides are generally accepted; they depend on the polysaccharide type, as well as on the water content [18,19,40]. For adhesion, different types of bonds across the interface are also important, e.g., ionic, covalent or hydrogen bonds. Together, they contribute to “wet” adhesion, meaning they are activated by moisture, and can, therefore, adhere non-specifically to many surfaces [41]. After activation and subsequent dehydration, they show a strong adhesion to dry surfaces. Polysaccharides (pectins, hemicelluloses) in the mucilage envelope have characteristic branched structures containing hydroxyl groups that are able to form hydrogen bonds [4,9,42,43]. This can enable them to interact with the surface of the substrate and promote a strong bond. For example, the seeds of *Plantago ovata* produce copious mucilage with the presence of both a highly branched structure of arabinoxylan and side-chains rich in sites with potential hydrogen-bond formation that can influence the rheological properties of the mucilage [10]. After hydration, arabinoxylans solubilize themselves and form a viscoelastic liquid outer layer that undergoes gelation in water. This layer in turn provides a reliable protective viscoelastic shell around the seed [10] but is soft/fluid enough to establish strong contact with the substrate when it dries. In contrast, the mucilage of *P. lanceolata* contains the highest proportion of unsubstituted xylan backbone [36] and, therefore, has lower adhesion values when compared with *P. ovata*.

The specific composition of *L. usitatissimum* mucilage can also be responsible for its strong adhesive properties. Important components of flax mucilage are branched rhamnogalacturonan (RG I) and arabinoxylan; the mixture of these two polysaccharides is responsible for the viscous properties of the mucilage in this plant species [43].

### 4.2. Cellulose Fibrils as an Additional Strengthening Component of the Mucilage Structure

The presence of cellulose fibrils can also be a factor in the maintenance of the seed envelope under mechanical loads. Cellulose fibrils allow for a more uniform distribution of mucilage around the seed and a stronger attachment to the seed surface -compare with data in [4]. The cellulose chains, composed of glucose residues, form elementary unbranched microfibrils [44,45,46,47,48], which can contain cellulose molecules that are highly ordered, and form crystal-like structures, in contrast to the unstructured, amorphous regions commonly seen. Hydroxyl groups in the crystalline phase form a large number of hydrogen bonds, which are responsible for the compact crystal structure of this region of the fibril. The free hydroxyls in the cellulose have a strong affinity to water, which can only be absorbed in the amorphous region of the fibril [47,49]. We observed in this study that cellulose fibrils can enhance the mechanical properties of mucilage. The maximal *F_ad_* measured during the detachment of the affixed seed from the glass surface was very high for the cellulose type of mucilage (Table 1). During the studies of the frictional properties of the critical-point dried cellulosic mucilage, we also detected adhesive forces caused by close contact of cellulose fibrils with the probe surface. We supposed that observed adhesive properties of mucilage can be based on van der Waals interactions between the cellulose fibers and the surface [30].

Cellulose mucilage of *O. basilicum* and *S. hispanica* demonstrated a specific lamellar structure. Such organization was observed in cellulose nanocomposite films obtained from microfibrillated cellulose (MFC) and amylopectin [50]. High tensile strength is combined with both high elasticity modulus and works of fracture in this nanocomposite. An elasticity modulus of 6.2 GPa, a tensile strength of 160 MPa and a strain-to-failure of 8.1% were observed at 70 wt. % of MFC reinforcement. The starch matrix used in this nanocomposite showed high compatibility with MFC, which aided in achieving the uniquely high MFC content. The amylopectin matrix of this nanocomposite is highly plasticized, which enables it to mimic the viscous properties in the cellulose “matrix” [50]. Only *O. basilicum* possesses starch grains in the mucilage. We can, therefore, conclude that the interaction between starch grains and cellulose (or other polysaccharide) fibrils can increase the adhesive properties measured in this mucilage.

### 4.3. Influence of the Contact Area and Mucilage Thickness on the Adhesive Properties

The differences between contact area measured for different seeds can result from the morphological features of the seeds and mucilage, such as the mass, size, and shape of the seed or the size (amount) of the mucilage envelope. The largest and most flattened seeds were characteristic of *L. usitatissimum,* but the contact area on the smooth glass surface was the smallest one in this species. High contact area was characteristic of *O. basilicum* and *P. ovata*, because these two taxa produce copious mucilage envelopes. In the natural habitat, all morphological seed features, including those of the mucilage, can play important roles in the seed attachment to the porous ground. It was observed that seed losses decreased with the increase in seed mass, and that the flat seeds had a higher resistance when removed from the soil surface. The presence of mucilage, which reduces susceptibility of seed removal by soil erosion, is an important mechanism for preventing further seed dispersal and maintaining the plant in its habitat [16].

Adhesion strength depends on the internal structure of the adhesive near the interface. The inclusion of other material in the glue matrix drastically influences adhesive strain energy density. While the adhesive strain energy density of the UHU glue was 36.5 mJ/m^2^, the adhesive strain energy density of husk mucilage of *P. ovata* seeds was just 2.74 mJ/m^2^. Such elastic properties of *P. ovata* husk mucilage can provide sufficient strength of the bond, preventing easy cohesive failure. The husk remnants might be responsible for the low strain energy density value. It is also possible to estimate the strain energy density value in a pull-off experiment on individual seeds. Assuming the same value of Young’s modulus for the mucilage in seeds as for the husk mucilage and taking the dry mucilage thickness of 3 µm (Figure 6G), the strain energy density value of a pure mucilage is 3 mJ/m^2^. This value is very close to that of the husk mucilage. We can estimate the strain energy density value in a pull-off experiment on seeds, assuming the same value of Young’s modulus and mucilage layer thickness. The strain energy density value for an individual seed, 21.6 mJ/m^2^, is one order of magnitude higher than that for the husk mucilage and close to the value for the UHU glue. Obviously, such elastic properties of *P. ovata* mucilage can provide sufficient strength of the bond, simultaneously preventing seed damage. Since the mucilage Young’s moduli for other species are unknown, we can compare the *W·E* values, which is an important parameter to characterize the adhesion efficiency. After normalization of the *W·E* value for *P. ovata* mucilage, it is possible to distinguish three groups with similar values. In the first group (*L. sativum*, *P. lanceolata*, husk mucilage and UHU glue), the *W·E* values are relatively small: 0.13–0.18. In the second group (*L. usitatissimum* and *S. hispanica*), the *W·E* values are higher: 0.57–0.64. Finally, *O. basilicum* has a *W·E* of 0.35, which is an intermediary between the first and second groups.

### 4.4. Adhesive Properties and Their Biological Significance

Diverse functional significances of adhesive or viscous properties of the diaspores are well recognized but studied only at the morphological level [13]. Nevertheless, the studies characterizing adhesive properties of the mucilage envelope with reference to its basic chemical composition, morphology and biological function are lacking.

From the biological point of view, strong adhesion can be extremely important, particularly in relation to long-distance diaspore dispersal. Mucilaginous seeds can adhere to an animal’s fur or a bird’s feathers. Fixed with the dried mucilage, they can then be transported over long distances [11,13,14,15]. Such a way of dispersal has been reported for mucilaginous seeds of several Euphorbia species, which were carried by birds between the Hawaiian Islands [11]. Similarly, the diaspores of *Lepidium sativum,* were dispersed, most likely by epizoochory, as was observed in New Zealand. Seabirds were likely the main dispersal vector to which the mucilaginous diaspores of diverse Lepidium taxa adhered, allowing them to be transported along various distances [51]. The example of the mucilaginous seeds of *Plantago media,* which were introduced from Europe to North America by traveling on human shoes and/or animal feet [5], demonstrates that the strong adhesive properties of the mucilage were the driving force in spreading this plant over the entire continent. Contrastingly, antitelochory has the opposite effect to diaspore dispersal, but adhesion also plays an important role here. Strong adhesion of the mucilaginous seeds to the soil protects them from being moved to less favorable habitats and enables germination in the surroundings of the mother plant [7].

Comparing our previous results on adhesion measurements of the hydrated mucilage envelope of *L. usitatissimum* (91 mN) and *P. lanceolata* (32 mN) [18,19] to the results obtained in the present study, we can differentiate between hydrated and dried mucilage adhesive properties. In the first case, low frictional and adhesive properties can be important for hydrated mucilage, in order to form the first contact between the seed and the substrate surface (ground, animal). In the second phase, dried-out mucilage, when the seed is strongly cemented to the surface, can be responsible for the long-distance dispersal of diaspores.

In conclusion, the results obtained demonstrated strong adhesive properties of the dried seed mucilage. The differences in measured variables depended on the mucilage type and, particularly, on the presence of a complex cellulose skeleton and/or highly branched polysaccharides (pectins, hemicelluloses), which are able to form adhesion-supporting hydrogen bonds. These results provide important information for the fields of seed biology and plant ecology.

Since the adhesive strength of the mucilage envelope can reach significantly higher values in comparison with the standard type of commercial glues, it has significant potential for the development of novel environment-friendly, non-toxic adhesives with strong bonding properties.

## Figures and Tables

**Table 1:** Results of pull-off forces measurements. Data on forces obtained with mucilaginous seeds and in complementary control tests with UHU glue.

Mucilage Type	Pectic		Cellulose						Hemicellulose			
Measurements	*Linum usitatissimum*	UHU	*Lepidium sativum*	UHU	*Ocimum basilicum*	UHU	*Salvia hispanica*	UHU	*Plantago lanceolata*	UHU	*Plantago ovata*	UHU
**Max *F_ad_***–adhesion force [N]:												
mean	1.01	3.82	2.83	1.65	4.44	1.37	1.59	1.79	1.30	1.41	3.15	2.3
max.	2.1	7.1	4.22	2.99	6.22	2.8	3.24	2.76	2.03	2.42	5.74	4.76
min.	0.62	1.48	1.24	0.76	2.88	0.55	0.89	0.37	0.58	0.2	0.91	1.17
SD	±0.43	±1.85	±0.82	±0.65	±0.85	±0.66	±0.56	±0.78	±0.4	±0.57	±1.47	±1.06
**Max *A*_0_**-contact area of mucilage envelope [mm^2^]:												
mean	0.98	5.4	4.09	1.96	3.22	1.51	1.57	0.86	1.10	1.58	3.35	2.15
max.	2.85	7.72	4.99	3.58	6.25	2.51	3.78	1.51	1.95	3.34	6.73	2.92
min.	0.35	2.17	2.9	0.34	1.35	0.36	0.11	0.07	0.55	0.24	0.59	0.78
SD	±0.70	±1.79	±0.57	±0.78	±1.26	±0.72	±1.32	±0.39	±0.40	±0.71	±1.9	±0.62
**Max *F_ad_*/*A*_0_**-adhesive strength [N/mm^2^]:												
mean	1.41	0.79	0.7	1.0	1.55	1.02	1.0	0.56	1.27	0.96	0.94	1.19
max.	3.53	2.07	1.02	2.4	2.68	1.53	8.15	1.45	2.27	1.85	0.85	3.42
min.	0.36	0.23	0.27	0.33	0.54	0.38	0.85	0.20	0.77	0.47	0.55	0.42
SD	±0.86	±0.48	±0.21	±0.59	±0.55	±0.37	±2.65	±0.37	±0.45	±0.41	±1.89	±0.75

## Data Availability

Not applicable.
